# Childhood Physical Abuse Casts a Very Long Shadow: Physical and Mental Illness Among Older Adults

**DOI:** 10.1093/geroni/igab046.1213

**Published:** 2021-12-17

**Authors:** Esme Fuller-Thomson, Anna S Buhrmann

**Affiliations:** 1 University of Toronto, Toronto, Ontario, Canada; 2 McMaster University, McMaster University, Hamilton, Ontario, Canada

## Abstract

A burgeoning literature indicates adverse childhood experiences (ACEs) are associated with chronic illness. Most research, to date, has not focused on health outcomes among older adults. The objectives of the current study were to identify the prevalence and adjusted odds of two mental health and six physical health conditions among survivors of childhood physical abuse (CPA) who were aged 60 and older (n=409) in comparison to their peers who had not been physically abused (n=4,659). Data were drawn from a representative sample of older British Columbians in the Canadian Community Health Survey. Logistic regression analyses took into account sex, race, age, immigration status, marital status, education, income, smoking, obesity, binge drinking and number of other ACEs. For 3 health outcomes, CPA survivors had adjusted odds ratio more than twice that of their peers (Anxiety OR=2.22; 95% CI=1.46, 3.38; Depression OR=2.17; 95% CI=1.57, 3.01; COPD OR=2.03; 95% CI=1.40, 2.94). For CPA survivors, the adjusted odds ratios were more than 50% higher for cancer (OR=1.71; 95% CI=1.31, 2.24), migraine (OR=1.67; 95% CI=1.15, 2.45) and debilitating chronic pain (OR=1.58; 95% CI=1.22, 2.03), and 33% higher for arthritis (OR=1.33; 95% CI=1.05, 1.69). CPA was not significantly associated with either heart disease or diabetes (p>.05). The association between CPA and two mental health and four physical health outcomes remained significant, even after controlling for sociodemographic characteristics, health behaviors and other ACEs. Further research is needed to investigate potential pathways through which childhood physical abuse is linked to a wide range of chronic later-life health problems.

